# TAM-derived extracellular vesicles containing microRNA-29a-3p explain the deterioration of ovarian cancer

**DOI:** 10.1016/j.omtn.2021.05.011

**Published:** 2021-05-19

**Authors:** Lili Lu, Wanwen Ling, Zhengyi Ruan

**Affiliations:** 1 Department of Obstetrics and Gynecology, Shanghai Ninth People’s Hospital, School of Medicine, Shanghai Jiaotong University, No. 639, Zhizaoju Road, Huangpu District, Shanghai 200011, PRC China

**Keywords:** tumor-associated macrophage-derived extracellular vesicles, microRNA-29a-3p, forkhead box protein O3, serine/threonine protein kinase/glycogen synthase kinase 3β, programmed death ligand-1, ovarian cancer, immune escape

## Abstract

Extracellular vesicles (EVs) secreted from tumor-associated macrophages (TAMs) are known to generate an immune-suppressive environment conducive to the development of ovarian cancer (OC). We tried to elucidate the role of TAM-derived exosomal microRNA (miR)-29a-3p in OC. miR-29a-3p, forkhead box protein O3 (FOXO3), and programmed death ligand-1 (PD-L1) expression was determined and their interactions evaluated. EVs were isolated, followed by determination of the uptake of EVs by OC cells, after which the proliferation and immune escape facilities of the OC cells were determined. OC xenograft models were constructed with EVs in correspondence with *in vivo* experiments. Overexpressed miR-29a-3p was detected in OC, and miR-29a-3p promoted OC cell proliferation and immune escape. EVs derived from TAMs enhanced the proliferation of OC cells. miR-29a-3p was enriched in TAM-EVs, and TAM-EVs delivered miR-29a-3p into OC cells. Downregulated FOXO3 was identified in OC, whereas miR-29a-3p targeted FOXO3 to suppress glycogen synthase kinase 3β (GSK3β) activity via the serine/threonine protein kinase (AKT)/GSK3β pathway. Inhibition of TAM-derived exosomal miR-29a-3p decreased PD-L1 to inhibit OC progression through the FOXO3-AKT/GSK3β pathway *in vitro* and *in vivo*. Taken together, the current studies highlight the FOXO3-AKT/GSK3β pathway and the mechanism by which TAM-derived exosomal miR-29a-3p enhances the expression of PD-L1 to facilitate OC cell proliferation and immune escape.

## Graphical abstract

**Figure:**
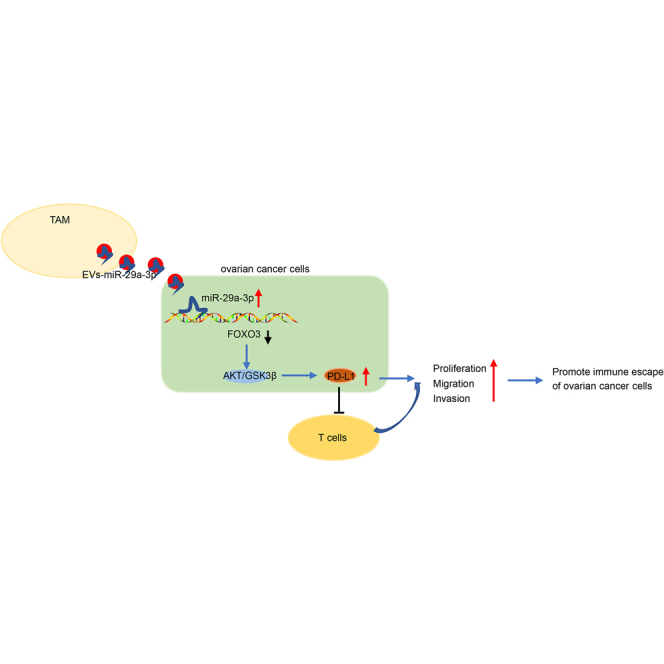

## Teaser

We found overexpressed miR-29a-3p in OC and found that miR-29a-3p shuttled by TAM-EVs promoted OC cell proliferation and immune escape. IN addition, miR-29a-3p targeted FOXO3 to suppress GSK3β activity through the AKT/GSK3β pathway. Altogether, our study uncovered a new FOXO3-AKT/GSK3β pathway by which TAM-derived exosomal miR-29a-3p enhanced PD-L1 expression to facilitate OC progression.

## Introduction

Gynecological cancers represent a chief cause of cancer-associated deaths on a global scale.^1^ Ovarian cancer (OC) remains a significant cause of malignancy afflicting the female reproductive tract, capable of inflicting considerable morbidity, and is associated with significant mortality.^2^^,^^3^ Currently, there is a lack of diagnostic markers in early stages and drug resistance often advances after anti-OC therapy, which are the two most primary barriers to the treatment of OC, especially when the cancer progresses to advanced stages.^4^ Existing literature has suggested that extracellular vesicles (EVs), a group of membrane-bound vesicles that are secreted from multiple kinds of cells, not only play vital roles in intercellular communications by effectively delivering lipids, proteins, and various nucleic acids but also have emerged as a novel therapeutic platform exhibiting a promising potential to treat various types of diseases including cancer.^5^ Previous reports have highlighted the involvement of exosomal microRNAs (miRNAs) released from macrophages in the development of drug resistance in epithelial OC.^6^ However, the functions of exosomal miRNAs in the development of OC remain somewhat controversial. Notably, miR-29a-3p has been demonstrated to be enriched in the exosomes derived from tumor-associated macrophages (TAMs), with studies indicating that it functions as a vital regulator for T cell differentiation in OC.^7^ Based on the aforementioned exploration of literature, we asserted the assumption that TAM-derived exosomal miR-29a-3p could regulate immunity to affect the progression of OC.

Intriguingly, another miRNA, miR-590-3p, has been reported to target forkhead box protein O3 (FOXO3) to facilitate invasiveness and proliferation of OC cells.^8^ Through microarray analysis, a binding site between miR-29a-3p and FOXO3 was predicted, inferring a similar targeting relationship between miR-29a-3p and FOXO3. Accumulating evidence continues to implicate the downregulation of miR-29a with the upregulation of FOXO3A,^9^ while depleted FOXO3 expression has been linked with unfavorable OC prognosis.^10^ Thus, miR-29a-3p may accelerate the development of OC by targeting FOXO3. FOXO3 is known as a tumor-inhibitive transcriptional factor and interacts with serine/threonine protein kinase (AKT) in OC.^11^ Shen et al. asserted the notion that miR-29a contributes to the drug resistance observed in breast cancer cells through the AKT/glycogen synthase kinase 3β (GSK3β) pathway.^12^ Importantly, the AKT/GSK3β signaling pathway represents one of the most notably altered entities in OC.^13^ Furthermore, inhibition of GSK3β activity upregulates the expression of programmed death ligand 1 (PD-L1) and ultimately inhibits T cell activity, thereby leading to immune escape.^14^ Hence, we asserted the hypothesis that miR-29a-3p could target FOXO3 expression to activate the AKT/GSK3β pathway, thereby promoting OC cell proliferation and contributing to immune escape.

## Results

### miR-29a-3p is highly expressed in OC and correlates with poor prognosis of OC patients

**Figure 1:**
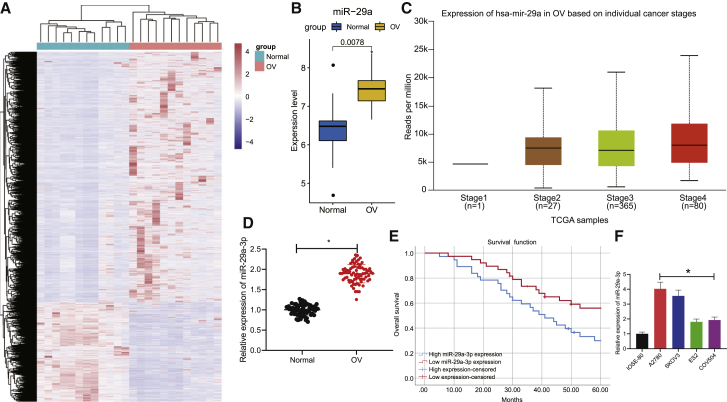
miR-29a-3p is overexpressed in OC and is associated with poor prognosis of OC patients (A) Heatmap of differentially expressed genes in GSE14407 microarray, where each row represents a differentially expressed gene and each column represents a sample. (B) The expression of miR-29a in GSE14407 microarray (Normal indicates normal tissues and OC indicates OC tissues in abscissa). (C) The expression of miR-29a-3p in OC tissues and tumor-adjacent tissues in The Cancer Genome Atlas(TCGA). (D) The expression of miR-29a-3p in OC tissues and tumor-adjacent tissues detected by qRT-PCR; n = 75. (E) Survival analysis was performed by Kaplan-Meier method (red line indicates high survival, and blue line indicates low survival). (F) The expression of miR-29a-3p in OC cell lines (A2780, SKOV3, ES2, and COV504) and IOSE80 cell line detected by qRT-PCR. ∗p < 0.05.

Following the differential analysis of the microarray dataset GSE14407, we found that 6,480 genes were upregulated and 2,584 genes were downregulated in OC patients compared with the normal control samples (Figure 1A) while miR-29a was upregulated in OC patient tissues (Figure 1B). We found from the UALCAN website that miR-29a is highly expressed in OC tissues compared with normal tissues (Figure 1C). Quantitative reverse transcriptase polymerase chain reaction (qRT-PCR) was performed and provided verification attesting that the expression of miR-29a-3p was higher in the OC tissues (Figure 1D). Survival analysis by Kaplan-Meier method revealed that overall survival of OC patients with low miR-29a-3p expression was much higher than overall of these patients with high miR-29a-3p expression (Figure 1E). qRT-PCR revealed that the expression of miR-29a-3p in OC cell lines (A2780, SKOV3, ES2, and COV504) was markedly elevated compared with the IOSE80 cell line. The expression of miR-29a-3p in A2780 and SKOV3 cell lines was notably higher than that in ES2 and COV504 cell lines (Figure 1F); thus the A2780 and SKOV3 cell lines were selected for subsequent experiments. Altogether, the key findings suggested that miR-29a-3p was expressed at high levels in OC tissues and cells, while high expression levels of miR-29a-3p were linked with poor prognosis in patients with OC.

### Downregulation of miR-29a-3p inhibits OC cell proliferation and immune escape

**Figure 2:**
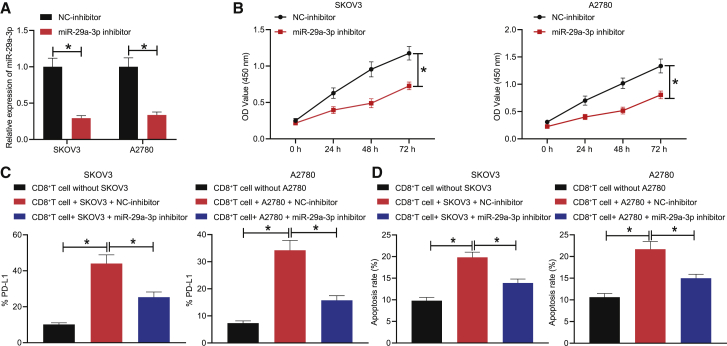
Downregulation of miR-29a-3p inhibits proliferation and immune escape of SKOV3/A2780 cells (A) The expression of miR-29a-3p in SKOV3 and A2780 cells after treatment with miR-29a-3p inhibitor determined by qRT-PCR. (B) The proliferation ability of SKOV3 and A2780 cells after treatment with miR-29a-3p inhibitor assessed by CCK8 assay. (C) The PD-L1 expression in CD8^+^ T cells co-cultured with SKOV3 or A2780 cells after treatment with miR-29a-3p inhibitor detected by flow cytometry. (D) The apoptosis rate of CD8+ T cells co-cultured with SKOV3 or A2780 cells after treatment with miR-29a-3p inhibitor detected by flow cytometry. ∗p < 0.05.

Next, to investigate the effects of miR-29a-3p on the proliferation and immune escape of OC cells, SKOV3 and A2780 cells were treated with negative control (NC)-inhibitor and miR-29a-3p inhibitor, respectively, with the expression of miR-29a-3p in each group of cells detected by qRT-PCR. The results obtained provided indication that expression of miR-29a-3p was markedly diminished in the SKOV3 and A2780 cells treated with miR-29a-3p inhibitor (Figure 2A). Cell counting kit8 (CCK8) results indicated that downregulation of miR-29a-3p markedly inhibited the proliferation of SKOV3 and A2780 cells (Figure 2B). Previous literature has suggested that reduction or apoptosis of CD8^+^ T cells contributes to immune escape.^15^^,^^16^ To evaluate the immune escape of OC cells, CD8^+^ T cells were co-cultured with the above-treated SKOV3 or A2780 cells. Flow cytometry was performed to detect the expression of PD-L1 in CD8^+^ T cells as well as the CD8^+^ T cell apoptosis rate. The results suggested that the expression of PD-L1 was increased in the CD8^+^ T cells co-cultured with SKOV3 or A2780 cells relative to that of the CD8^+^ T cells cultured alone and the rate of apoptosis was also elevated; in the co-culture system, PD-L1 expression was reduced in the CD8^+^ T cells upon miR-29a-3p inhibitor treatment and the apoptosis rate was also downregulated (Figures 2C and 2D). Thus, decreased miR-29a-3p was determined to inhibit the proliferation and immune escape of OC cells.

### miR-29a-3p in OC cells is derived from EVs of TAM (TAM-EVs)

**Figure 3:**
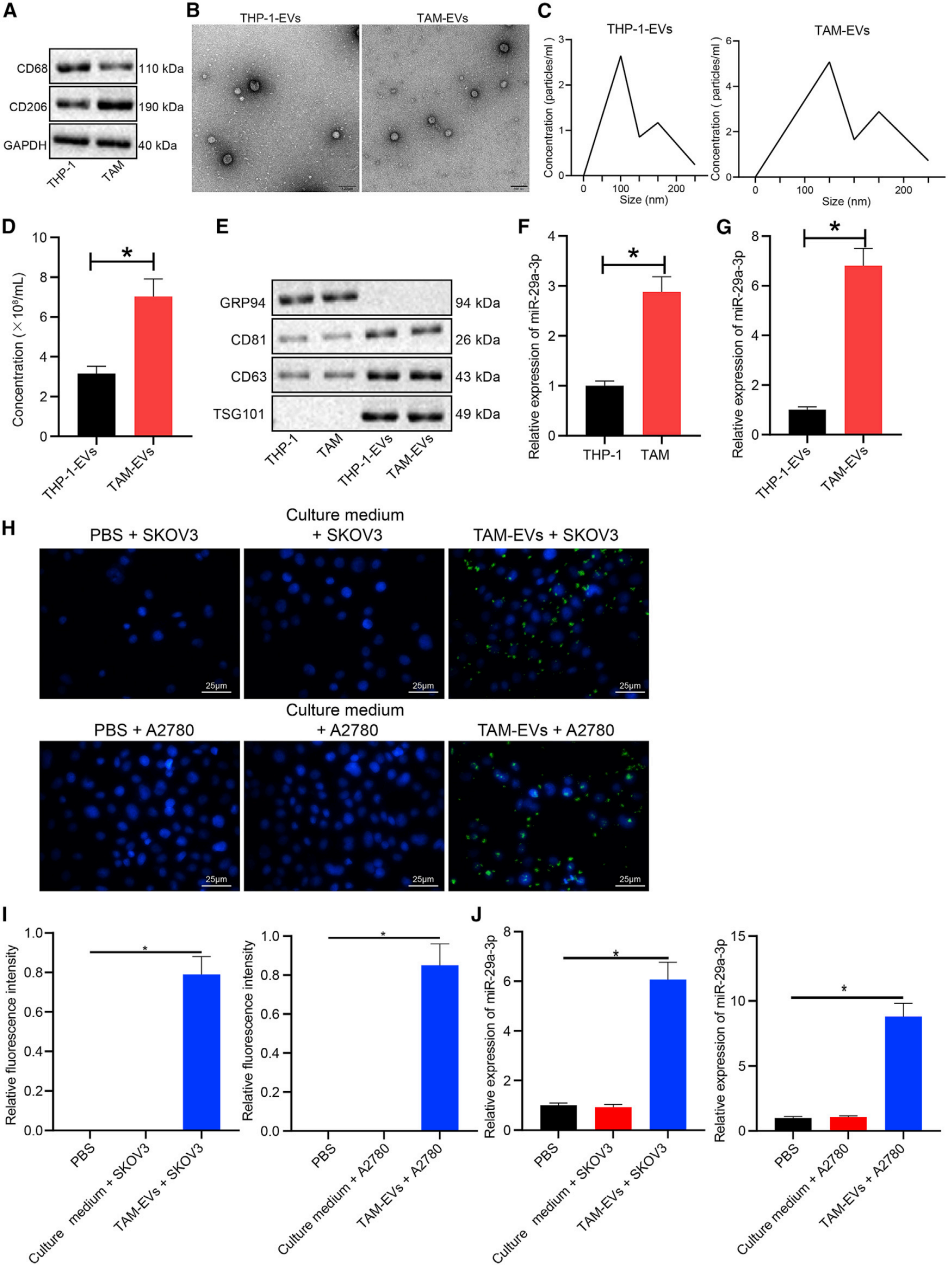
miR-29a-3p in SKOV3/A2780 cells comes from TAM-EVs (A) The protein expression of macrophage marker genes CD68 and CD206 in THP-1 cells and TAMs assessed by western blot normalized to GAPDH. (B) The structure diagram of THP-1-EVs and TAM-EVs identified by transmission electron microscopy (scale bars, 100 nm). (C) The diameter of THP-1-EVs and TAM-EVs detected by NanoSight tracking analysis. (D) The particle concentration of THP-1-EVs and TAM-EVs detected by NanoSight tracking analysis. (E) The protein expression of TSG101, CD63, CD81, and GRP94 in THP-1 cells and TAMs and their respective EVs measured by western blot. (F) The expression of miR-29a-3p in THP-1 cells and TAMs measured by qRT-PCR. (G) The expression of miR-29a-3p in THP-1-EVs and TAM-EVs measured by qRT-PCR. (H) The absorption of PKH67-labeled TAM-EVs by SKOV3 and A2780 cells observed by fluorescence microscopy, where the nucleus showed blue fluorescence and TAM-EVs showed green fluorescence (scale bars, 25 μm). (I) The content of Cy3-miR-29a-3p in SKOV3 and A2780 cells co-cultured with PBS, culture medium, or TAM-EVs. (J) qRT-PCR was used to detect the expression of miR-29a-3p in SKOV3 and A2780 cells treated with PBS, culture medium, or TAM-EVs. ∗p < 0.05.

Next, to ascertain whether TAM-EVs could deliver miR-29a-3p into OC cells, first, western blot was performed to determine the protein expression of macrophage marker genes (CD68 and CD206) in THP-1 cells and TAMs, and the results displayed lower protein expression of CD68 in TAMs than in THP-1 cells and higher protein expression of CD206 in TAMs than in THP-1 cells (Figure 3A). Next, the EVs of THP-1 cells and TAMs were extracted and observed by transmission electron microscopy, with the greater majority of the EVs observed to be round or oval in shape (Figure 3B). NanoSight particle tracking analysis of the diameter and particle concentration of EVs indicated that the EVs of THP-1 cells (THP-1-EVs) and TAM-EVs obtained generally ranged between 10 and 250 nm in diameter, with no significant difference detected between the two; however, the concentration of extracted TAM-EVs was higher than that of THP-1-EVs (Figures 3C and 3D). Western blot was performed to detect the expression of EV-specific marker proteins (CD63, CD81, and TSG101) and an endoplasmic reticulum marker (GRP94), with the results suggesting that TSG101, CD63, and CD81 protein expression was significantly elevated in THP-1-EVs and TAM-EVs relative to that in the THP-1 cells and TAMs whereas GRP94 was not expressed in THP-1-EVs and TAM-EVs (Figure 3E). The aforementioned findings confirmed the successful isolation of EVs. qRT-PCR provided evidence demonstrating that miR-29a-3p in THP-1 cells, TAMs, and their respective EVs revealed that miR-29a-3p expression was significantly increased in the TAMs compared with the THP-1 cells (Figure 3F) and miR-29a-3p expression was significantly increased in the TAM-EVs compared with the THP-1-EVs (Figure 3G). Next, to ascertain whether TAM-EVs could enter SKOV3 and A2780 cells, we labeled TAM-EVs with PKH67 and observed the absorption of TAM-EVs by SKOV3 and A2780 cells treated with the culture medium supplemented with 90% Roswell Park Memorial Institute (RPMI) 1640 medium + 10% fetal bovine serum (FBS), phosphate-buffered solution (PBS), or TAM-EVs. The results obtained indicated that there was no green fluorescence in SKOV3 and A2780 cells treated with PBS and the culture medium and strong green fluorescence was observed in the cytoplasm of SKOV3 and A2780 cells treated with TAM-EVs, suggesting that TAM-EVs were successfully engulfed into SKOV3 and A2780 cells (Figure 3H). Next, miR-29a-3p in TAM-EVs was labeled with Cy3, and the content of Cy3-miR-29a-3p in each group was determined by fluorescence microscopy. The results revealed red fluorescence (indicating no Cy3-miR-29a-3p) in SKOV3 and A2780 cells treated with PBS and the culture medium and red fluorescence (indicating high content of Cy3-miR-29a-3p) in >75% of SKOV3 and A2780 cells treated with TAM-EVs (Figure 3I). qRT-PCR revealed that the expression of miR-29a-3p in SKOV3 and A2780 cells was significantly elevated after treatment with TAM-EVs compared with PBS or culture medium treatment (Figure 3J). Taken together, miR-29a-3p in SKOV3/A2780 cells was derived from TAM-EVs.

### miR-29a-3p targets FOXO3

**Figure 4:**
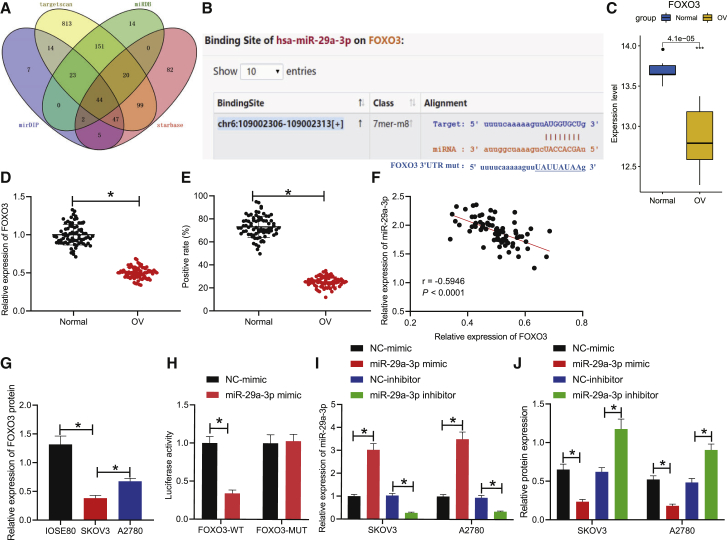
FOXO3 is a target gene of miR-29a-3p (A) Collection of miRNAs targeting FOXO3 predicted by miRDB, mirDIP, starBase, and TargetScan databases. (B) Binding sites between miR-29a-3p and FOXO3 predicted by TargetScan database. (C) The expression of FOXO3 in the microarray (Normal in abscissa indicates tumor-adjacent tissues; OC indicates OC tissues). (D) The transcription level of FOXO3 in OC tissues and tumor-adjacent tissues detected by qRT-PCR. (E) The positive expression of FOXO3 protein in OC tissues and tumor-adjacent tissues assessed by IHC. (F) Pearson’s correlation analysis between FOXO3 expression and miR-29a-3p level. (G) The protein expression level of FOXO3 in IOSE80, SKOV3, and A2780 cell lines detected by western blot normalized to GAPDH. (H) The targeting relationship between miR-29a-3p and FOXO3 assessed by dual-luciferase reporter gene assay. (I) The expression of miR-29a-3p in SKOV3 and A2780 cells treated with miR-29a-3p mimic or miR-29a-3p inhibitor detected by qRT-PCR. (J) The protein expression of FOXO3 in SKOV3 and A2780 treated with miR-29a-3p mimic or miR-29a-3p inhibitor detected by western blot normalized to GAPDH. ∗p < 0.05.

Next, to identify the downstream genes of FOXO3, targeted miRNAs of FOXO3 were predicted in connection with the miRDB, mirDIP, starBase, and TargetScan databases. Forty-four miRNAs were found to target FOXO3 in all four databases (Figure 4A), in which miR-29a-3p demonstrated a targeting relationship with FOXO3 (Figure 4B). Previous literature has indicated that FOXO3 expression is downregulated in OC patient tissues,^8^^,^^10^ which was further verified by the microarray data in our study (Figure 4C). The expression of FOXO3 in OC tissues and tumor-adjacent tissues was detected by qRT-PCR and immunohistochemistry (IHC), respectively, with the results indicating that the expression of FOXO3 in OC tissues was markedly lower than that in the tumor-adjacent tissues (Figures 4D and 4E). Pearson’s correlation analysis of the relationship between FOXO3 and miR-29a-3p revealed that the expression of FOXO3 was significantly negatively correlated with miR-29a-3p level (p < 0.001) (Figure 4F). Western blot analysis results demonstrated that the protein expressions of FOXO3 in SKOV3 and A2780 cell lines were significantly lower than that in the IOSE80 cell line (Figure 4G). The targeting relationship between miR-29a-3p and FOXO3 was verified by dual-luciferase reporter gene assay. The results displayed that treatment with miR-29a-3p mimic significantly decreased luciferase activity in HEK293T cells co-transfected with FOXO3-WT (p < 0.05); however, treatment with miR-29a-3p mimic failed to elicit any significant changes in the luciferase activity of the HEK293T cells co-transfected with FOXO3-MUT (p > 0.05), suggesting that miR-29a-3p could target and bind to FOXO3 (Figure 4H). Next, the expression of miR-29a-3p was either overexpressed or downregulated in SKOV3/A2780 cells, with qRT-PCR subsequently conducted to detect the expression of miR-29a-3p in SKOV3/A2780 cells. The results revealed that the expression of miR-29a-3p was significantly increased after treatment with miR-29a-3p mimic, whereas the expression of miR-29a-3p was markedly reduced after treatment with miR-29a-3p inhibitor (Figure 4I). The expression of FOXO3 in SKOV3 and A2780 cells was detected by western blot, respectively, with the results indicating that overexpression of miR-29a-3p inhibited the expression of FOXO3 and downregulation of miR-29a-3p elevated the expression of FOXO3 (Figure 4J). Taken together, the results demonstrated that FOXO3 could be targeted by miR-29a-3p.

### Downregulation of miR-29a-3p inhibits proliferation and immune escape of OC cells by decreasing PD-L1 expression through FOXO3-AKT/GSK3β axis

**Figure 5:**
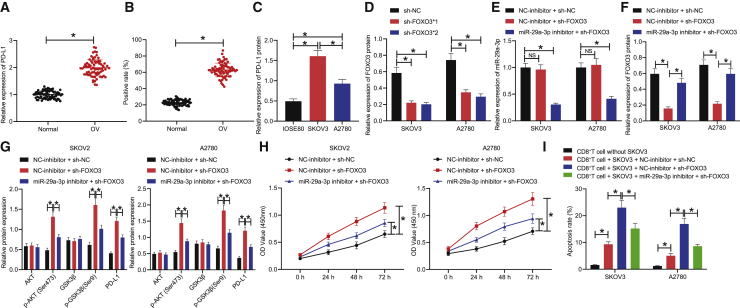
Inhibition of miR-29a-3p expression reduces PD-L1 expression through FOXO3-AKT/GSK3β axis, thus suppressing the proliferation of OC cells and immune escape (A) PD-L1 transcription level in OC tissues and tumor-adjacent tissues assessed by qRT-PCR. (B) PD-L1 protein positive expression in OC tissues and tumor-adjacent tissues measured by IHC. (C) PD-L1 protein expression in OC tissues and tumor-adjacent tissues measured by western blot normalized to GAPDH. (D) sh-FOXO3 sequences with the lowest FOXO3 expression screened by western blot normalized to GAPDH. (E) miR-29a-3p expression in SKOV3/A2780 cells treated with sh-FOXO3 or miR-29a-3p inhibitor assessed by qRT-PCR. (F) FOXO3 protein expression in SKOV3/A2780 cells treated with sh-FOXO3 or miR-29a-3p inhibitor assessed by western blot normalized to GAPDH. (G) The expression of AKT, p-AKT (Ser473), GSK3β, p-GSK3β (Ser9), and PD-L1 in SKOV3/A2780 cells treated with sh-FOXO3 or miR-29a-3p inhibitor measured by western blot normalized to GAPDH. (H) The proliferation ability of SKOV3/A2780 cells treated with sh-FOXO3 or miR-29a-3p inhibitor assessed by CCK8 assay. (I) The apoptosis rate of CD8^+^ T cells co-cultured with SKOV3/A2780 cells detected by flow cytometry. ∗p < 0.05.

The AKT/GSK3β pathway has been well documented as one of the most frequently altered pathways in OC. As a critical regulator in evading anti-tumor immunity, PD-L1 is often overexpressed in OC.^13^^,^^17^ To elucidate the interaction between them, qRT-PCR and IHC were performed to detect the expression of PD-L1 in OC tissues and tumor-adjacent tissues, respectively, with the results demonstrating that the expression of PD-L1 in OC tissues was much higher than that in tumor-adjacent tissues (Figures 5A and 5B). The protein expression of PD-L1 in each OC cell line was detected by western blot, with the results exhibiting significantly higher expression of PD-L1 in SKOV3 and A2780 cell lines relative to that in the IOSE80 cell line and lower expression of PD-L1 in the A2780 cell line than in the SKOV3 cell line (Figure 5C). Two FOXO3 silencing sequences, sh-FOXO3∗1 and sh-FOXO3∗2, were set up, followed by detection of their silencing efficiency by western blot. The results indicated that sh-FOXO3∗2 had the most distinct effect on reducing FOXO3 expression in SKOV3/A2780 cells (Figure 5D); thus, it was selected for subsequent experiments. SKOV3 and A2780 cells were treated with simultaneous downregulation of miR-29a-3p and inhibition of FOXO3 expression, after which the expression of miR-29a-3p, FOXO3, AKT, p-AKT (Ser473), GSK3β, p-GSK3β (Ser9), and PD-L1 in SKOV3 and A2780 cells was detected by qRT-PCR and western blot. The results revealed that treatment with NC-inhibitor + sh-FOXO3 brought about no significant difference in miR-29a-3p expression, along with significantly decreased FOXO3 level and markedly increased p-AKT (Ser473), p-GSK3β (Ser9), and PD-L1 levels; treatment of miR-29a-3p inhibitor + sh-FOXO3 significantly decreased miR-29a-3p level, increased FOXO3 level, and reduced p-AKT (Ser473), p-GSK3β (Ser9), and PD-L1 levels (Figures 5E–5G). Moreover, inhibition of FOXO3 alone or silencing of miR-29a-3p and FOXO3 brought about no significant difference in terms of the AKT and GSK3β levels (p > 0.05). Altogether, downregulation of miR-29a-3p was found to prevent activation of the AKT/GSK3β pathway by means of elevating the expression of FOXO3, and activation of GSK3β led to a decrease in the expression of PD-L1.

Next, to ascertain the effect of PD-L1 regulated by miR-29a-3p-FOXO3-AKT/GSK3β axis on OC cell proliferation and immune escape, CCK8 was initially performed to detect the cell proliferation ability of each group. The results revealed that the proliferation of SKOV3 and A2780 cells was increased after treatment with the NC-inhibitor + sh-FOXO3, whereas the proliferation of SKOV3 and A2780 cells was decreased after treatment of miR-29a-3p inhibitor + sh-FOXO3, implying that downregulation of miR-29a-3p inhibited OC cell proliferation ability (Figure 5H). After co-culture of CD8^+^ T cells with SKOV3 and A2780 cells, the co-culture system was treated as above. The rate of apoptosis was detected by means of flow cytometry, with the results indicating that the apoptosis rate of the CD8^+^ T cells co-cultured with the SKOV3/A2780 cells was increased compared with that of the CD8^+^ T cells cultured alone. In the SKOV3/A2780 co-culture system, apoptosis rate of CD8^+^ T cells was increased by treatment with NC-inhibitor + sh-FOXO3, but apoptosis rate was decreased in CD8^+^ T cells treated with miR-29a-3p inhibitor + sh-FOXO3, indicating that downregulation of miR-29a-3p inhibited CD8^+^ T cell apoptosis (Figure 5I). Taken together, depletion of miR-29a-3p was observed to trigger a decrease in the expression of PD-L1 via the FOXO3-AKT/GSK3β axis and inhibited the proliferation and immune escape of OC cells.

### Depletion of TAM-derived exosomal miR-29a-3p downregulates the expression of PD-L1 through the FOXO3-AKT/GSK3β axis, thus repressing the proliferation and immune escape of OC cells

**Figure 6:**
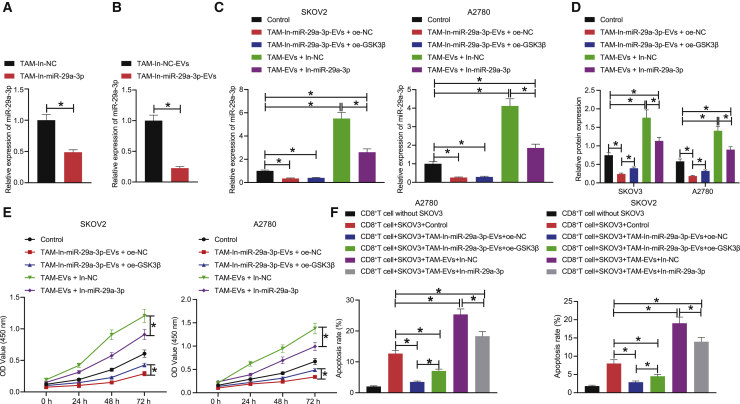
Silencing of TAM-derived exosomal miR-29a-3p can regulate the FOXO3-AKT/GSK3β axis to downregulate the expression of PD-L1, thereby inhibiting the proliferation and immune escape of OC cells (A) The expression of miR-29a-3p expression in TAMs treated with In-miR-29a-3p detected by qRT-PCR. (B) The expression of miR-29a-3p expression in EVs of TAMs treated with In-miR-29a-3p detected by qRT-PCR. (C) The expression of miR-29a-3p in SKOV3 and A2780 cells treated with TAM-In-miR-29a-3p-EVs, oe-GSK3β, or In-miR-29a-3p detected by qRT-PCR. (D) The expression of PD-L1 in SKOV3 and A2780 cells treated with TAM-In-miR-29a-3p-EVs, oe-GSK3β, or In-miR-29a-3p detected by western blot. (E) The proliferation ability of SKOV3 and A2780 cells treated with TAM-In-miR-29a-3p-EVs, oe-GSK3β, or In-miR-29a-3p detected by CCK8. (F) The apoptosis rate of T cells after co-culture of CD8^+^ T cells with SKOV3 and A2780 cells detected by flow cytometry. ∗p < 0.05.

Next, to evaluate the effect associated with the inhibition of TAM-derived exosomal miR-29a-3p on OC cell proliferation and immune escape through the FOXO3-AKT/GSK3β/PD-L1 axis, we infected TAMs with lentiviruses In-NC and In-miR-29a-3p and extracted their EVs, after which qRT-PCR was performed to detect the expression of miR-29a-3p in TAMs and their EVs in each group. Our results revealed that the expression of miR-29a-3p was significantly decreased in the TAMs infected with lentivirus expressing In-miR-29a-3p as well as in their respective EVs (Figures 6A and 6B). We subsequently set out to treat SKOV3/A2780 cells with TAM-In-miR-29a-3p-EVs + oe-NC, TAM-In-miR-29a-3p-EVs + oe-GSK3β, TAM-EVs + In-NC, or TAM-EVs + In-miR-29a-3p, followed by determination of the expression of miR-29a-3p and PD-L1. The results demonstrated that the miR-29a-3p and PD-L1 levels were markedly diminished in the SKOV3/A2780 cells treated with TAM-In-miR-29a-3p-EVs + oe-NC or TAM-In-miR-29a-3p-EVs + oe-GSK3β; miR-29a-3p and PD-L1 levels were notably elevated in the SKOV3/A2780 cells treated with TAM-EVs + In-NC or TAM-EVs + In-miR-29a-3p; whereas the miR-29a-3p levels were not significantly different with the PD-L1 levels observed to significantly increase in the SKOV3/A2780 cells treated with TAM-In-miR-29a-3p-EVs + oe-GSK3β relative to the SKOV3/A2780 cells treated with TAM-In-miR-29a-3p-EVs + oe-NC; miR-29a-3p and PD-L1 levels were significantly decreased in the SKOV3/A2780 cells treated with TAM-EVs + In-miR-29a-3p compared with the SKOV3/A2780 cells treated with TAM-EVs + In-NC (Figures 6C and 6D). Altogether, inhibition of TAM-derived exosomal miR-29a-3p was found to prevent AKT/GSK3β pathway activation by means of elevating the expression of FOXO3, ultimately enhancing GSK3β activity and downregulating PD-L1 expression.

The CCK8 results demonstrated that the proliferation of SKOV3 and A2780 cells was reduced after treatment with TAM-In-miR-29a-3p-EVs + oe-NC or TAM-In-miR-29a-3p-EVs + oe-GSK3β, and the proliferation of SKOV3 and A2780 cells was stimulated after treatment with TAM-EVs + In-NC or TAM-EVs + In-miR-29a-3p; the proliferation of SKOV3 and A2780 cells was increased after treatment with TAM-In-miR-29a-3p-EVs + oe-GSK3β compared with after treatment of TAM-In-miR-29a-3p-EVs + oe-NC; the promotive effect of TAM-EVs on the proliferation of SKOV3 and A2780 cells could be reversed via the downregulation of miR-29a-3p expression (Figure 6E). After co-culture of the CD8^+^ T cells with SKOV3/A2780 cells, the co-culture system was treated in an identical fashion to the aforementioned method. The apoptosis rate of the CD8^+^ T cells was detected by flow cytometry, the results of which indicated that the apoptosis rate of the CD8^+^ T cells was elevated in the CD8^+^ T cells co-cultured with SKOV3 and A2780 cells compared with that of the CD8^+^ T cells cultured alone. In the SKOV3/A2780 co-culture system, the apoptosis rate of the CD8^+^ T cells was decreased in the CD8^+^ T cells treated with TAM-In-miR-29a-3p-EVs + oe-NC or TAM-In-miR-29a-3p-EVs + oe-GSK3β and increased in CD8^+^ T cells treated with TAM-EVs + In-NC or TAM-EVs + In-miR-29a-3p; the apoptosis rate of the CD8^+^ T cells was increased in the CD8^+^ T cells treated with TAM-In-miR-29a-3p-EVs + oe-GSK3β compared with the CD8^+^ T cells treated with TAM-In-miR-29a-3p-EVs + oe-NC; downregulation of miR-29a-3p inhibited the apoptosis rate of the CD8^+^ T cells that was elevated by treatment with TAM-EVs + In-NC (Figure 6F). In summary, inhibition of TAM-derived exosomal miR-29a-3p was found to impede the proliferation and immune escape of OC cells by regulating the FOXO3-AKT/GSK3β axis to downregulate the expression of PD-L1.

### Downregulation of TAM-derived exosomal miR-29a-3p regulates FOXO3-AKT/GSK3β axis to inhibit PD-L1 expression, thereby inhibiting tumor formation and immune escape *in vivo*

**Figure 7:**
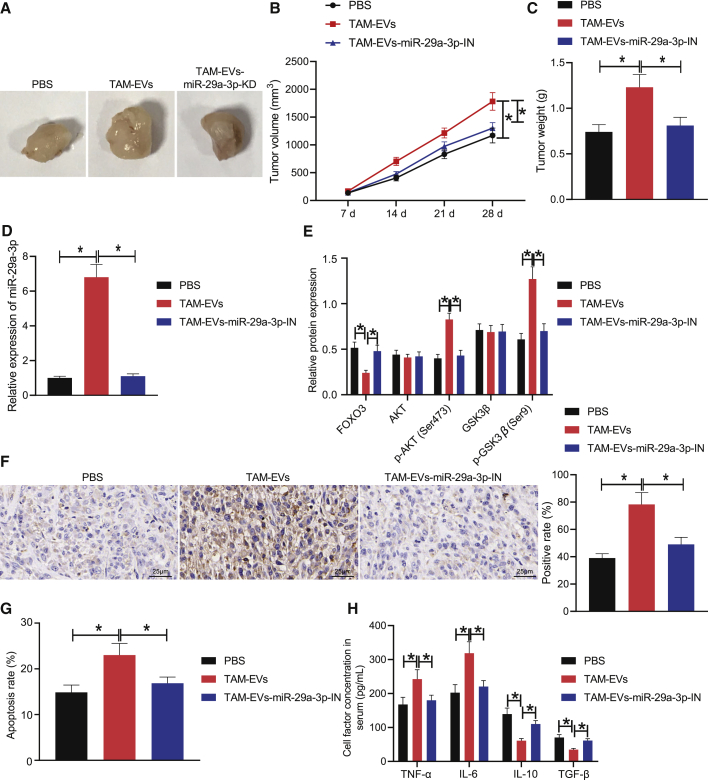
Inhibited TAM-derived exosomal miR-29a-3p can inhibit PD-L1 expression through FOXO3-AKT/GSK3β axis, thereby repressing tumor formation and immune escape *in vivo* (A) Anatomical maps of tumors of mice treated with PBS, TAM-EVs, or TAM-In-miR-29a-3p-EVs. (B) Tumor volume of mice treated with PBS, TAM-EVs, or TAM-In-miR-29a-3p-EVs. (C) Tumor weight of mice treated with PBS, TAM-EVs, or TAM-In-miR-29a-3p-EVs. (D) The expression of miR-29a-3p in serum EVs of mice treated with PBS, TAM-EVs, or TAM-In-miR-29a-3p-EVs detected by qRT-PCR (cel-miR-39 was used as an internal reference). (E) The expression of FOXO3, AKT, p-AKT (Ser473), GSK3β, and p-GSK3β (Ser9) in tissues of mice treated with PBS, TAM-EVs, or TAM-In-miR-29a-3p-EVs detected by western blot. (F) The expression of PD-L1-positive protein in tumor tissues of mice treated with PBS, TAM-EVs, or TAM-In-miR-29a-3p-EVs assessed by IHC (scale bars, 25 μm). (G) The PD-L1 expression in CD8^+^ T cells in spleen tissues of mice treated with PBS, TAM-EVs, or TAM-In-miR-29a-3p-EVs detected by flow cytometry. (H) The content of immune factors (TNF-α, IL-6, IL-10, TGF-β) in the serum of mice treated with PBS, TAM-EVs, or TAM-In-miR-29a-3p-EVs detected by ELISA. ∗p < 0.05.

To elucidate the aforementioned mechanism *in vivo*, we subcutaneously inoculated SKOV3 cells in nude mice to establish OC xenograft models and injected the model mice with PBS, TAM-EVs, and TAM-In-miR-29a-3p-EVs. The xenograft growth curve and weight results revealed that the tumor growth was accelerated and the tumor weight was increased in the TAM-EV-treated mice relative to those of the PBS-treated mice; tumor growth was slowed and tumor weight was decreased in the TAM-In-miR-29a-3p-EV-treated mice compared with TAM-EV-treated mice (Figures 7A–7C). The expression of miR-29a-3p in the EVs of serum cells detected by qRT-PCR revealed that the expression of miR-29a-3p was significantly increased in the TAM-EV-treated mice compared with the PBS-treated mice; the miR-29a-3p expression was markedly decreased in the TAM-In-miR-29a-3p-EV-treated mice compared with TAM-EV-treated mice (Figure 7D). Western blot analysis of the expression of FOXO3 protein and AKT/GSK3β pathway-related proteins in tumor tissues of nude mice revealed that the FOXO3 level was significantly decreased while the p-AKT (Ser473) and p-GSK3β (Ser9) levels were significantly increased in the TAM-EV-treated mice relative to those of the PBS-treated mice; the FOXO3 level was significantly increased while the p-AKT (Ser473) and p-GSK3β (Ser9) levels were significantly decreased in the TAM-In-miR-29a-3p-EV-treated mice compared with TAM-EV-treated mice (Figure 7E). There was no significant difference in the AKT and GSK3β levels after treatment with PBS, TAM-EVs, or TAM-In-miR-29a-3p-EVs.

Furthermore, IHC detection results of the positive expression of immune cell-associated protein PD-L1 in the tumor tissues of mice uncovered that the expression of PD-L1-positive protein was increased in the TAM-EV-treated mice compared with PBS-treated mice; the expression of PD-L1-positive protein was decreased in the TAM-In-miR-29a-3p-EV-treated mice compared with TAM-EV-treated mice (Figure 7F). Flow cytometry of PD-L1 expression in the CD8^+^ T cells in the splenic tissues of the mice indicated that the expression of PD-L1 in the CD8^+^ T cells was increased in the TAM-EV-treated mice compared with the PBS-treated mice; PD-L1 expression in CD8^+^ T cells was decreased in the TAM-In-miR-29a-3p-EV-treated mice compared with the TAM-EV-treated mice (Figure 7G). Finally, the contents of immune factors (tumor necrosis factor α [TNF-α], interleukin-6 [IL-6], interleukin-10 [IL-10], and transforming factor β [TGF-β]) in the serum of mice detected by enzyme-linked immunosorbent assay (ELISA) indicated that compared with the PBS-treated mice, the levels of TNF-α and IL-6 were significantly increased whereas the levels of IL-10 and TGF-β were significantly decreased in the TAM-EV-treated mice; the levels of TNF-α and IL-6 were significantly decreased whereas the levels of IL-10 and TGF-β were significantly increased in the TAM-In-miR-29a-3p-EV-treated mice compared with the TAM-EV-treated mice (Figure 7H). Taken together, depletion of TAM-derived exosomal miR-29a-3p could lead to downregulation of PD-L1 expression via the FOXO3-AKT/GSK3β axis, thereby inhibiting tumor formation and immune escape *in vivo*.

## Discussion

The progression of OC is significantly influenced by the immune microenvironment, which is comprised of TAMs and T lymphocytes, with exosomes that typically regulate the interactions between TAMs and T cells to create an immune-inhibitive environment that stimulates OC development through miRNAs, including miR-29a-3p enriched in TAM-EVs.^7^^,^^18^ Thus, both exosomes and their relevant miRNAs represent promising therapeutic targets for OC treatment. During the current study, our results demonstrated that downregulation of TAM-derived exosomal miR-29a-3p regulated the FOXO3-AKT/GSK3β axis to inhibit PD-L1 expression, ultimately inhibiting OC cell proliferation and immune escape.

Previous literature has documented the causal role of miRNAs in the carcinogenesis of numerous human cancers including OC.^19^ Immune evasion adds an additional layer of complexity for the immune system’s ability to recognize and destroy cancer cells, creating a significant stumbling block in the development of effective therapeutic strategies for OC.^20^^,^^21^ Key observations made during the present study revealed that a high level of miR-29a-3p expression in OC was conducive to OC cell immune escape. Previous studies have highlighted miR-29a-3p as a member of the miR-29 family, suggesting that it plays a crucial role in T cell differentiation while flagging its ability to suppress the differentiation of CD4^+^ T cells.^22^^,^^23^ Elevated immune escape of tumor cells has been implicated in the suppression of intra-tumor T cell infiltration.^24^ The aforementioned evidence further highlights the role of miR-29a-3p in promoting the immune escape of OC cells.

Our findings provided evidence suggesting that TAM-EVs could deliver miR-29a-3p into OC cells and TAM-derived exosomal miR-29a-3p targeted FOXO3 to regulate OC development. Previous research has indicated the transfer of miRNAs via exosomes as a crucial mechanism involved in the exchange of genetic information between cells.^25^ EVs derived from macrophages have been previously reported to deliver miRNAs into OC cells,^6^ while TAM-EVs containing miR-29a-3p have been shown to regulate tumor immunity in OC,^7^ attesting to the role of miR-29a-3p in OC cells secreted from TAM-EVs, exacerbating OC. Similarly, TAM-derived exosomal miR-223 has been previously reported to promote a chemo-resistant phenotype in epithelial OC cells.^6^ FOXO3 has been previously demonstrated to be a direct target gene of miR-29a-3p.^9^ In addition, low expression of FOXO3 was found in OC tissues.^10^ Moreover, miR-29a-3p released from TAM-EVs has been speculated to aid in facilitating the progression of OC by means of targeting FOXO3. Similarly, it was demonstrated that miR-590-3p accelerates proliferation and spheroid formation of OC cells by targeting FOXO3.^8^

Furthermore, our data suggested that miR-29a-3p could regulate the AKT/GSK3β axis that enhanced the expression of PD-L1, which ultimately stimulated the proliferation and immune escape capacity of the OC cells. Previous evidence has provided verification of FOXO3 as a target gene of miR-29a-3p, and as a tumor-inhibitive transcriptional factor FOXO3 has been demonstrated to suppress the development of OC through its interactions with AKT.^11^ Notably, the AKT/GSK3β signaling pathway is generally activated in OC, with the activation of AKT shown to inactivate GSK-3β through phosphorylation at Ser9, whereby the phosphorylation site of AKT is Ser473 (AKT activation), and the phosphorylation site of GSK3β is Ser9 (GSK3β inactivation).^13^^,^^26^ The inhibition of GSK3β activity has been reported to elevate the expression of PD-L1, which suppresses T cell activity and consequently contributes to immune escape.^14^ As a key immune molecule that negatively regulates immune responses and an important marker of immune escape, PD-L1 has been highlighted as a critical downstream oncogenic component of AKT3 in OC cells and is overexpressed in OC cells.^17^^,^^27^ Studies have also shown that reduction or apoptosis of CD8^+^ T cells can lead to immune escape.^16^ Additionally, PD-L1 expression was negatively correlated with CD8^+^ T cell counts and inhibited the function of CD8^+^ T cells.^15^ Likewise, Schachtele et al.^28^ proposed that CD8^+^ T lymphocytes are negatively regulated by PD-L1 in viral encephalitis, which was partially consistent with the observations of our study. The aforementioned findings were also established in mouse models of OC, providing *in vivo* evidence for the function of TAM-released exosomal miR-29a-3p in OC progression. In summary, TAM-derived exosomal miR-29a-3p mediates the FOXO3-AKT/GSK3β axis to enhance PD-L1 expression, consequently promoting OC cell proliferation and immune escape *in vitro* and *in vivo*.

**Figure 8:**
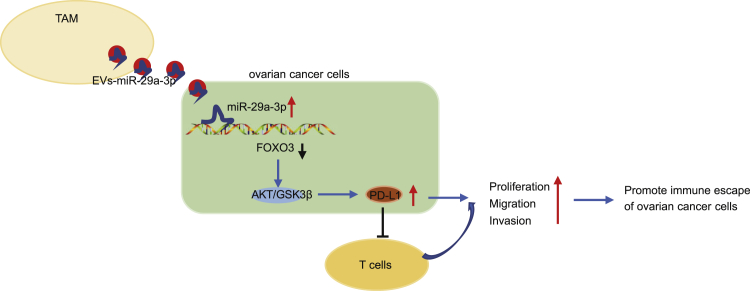
Schematic map of the regulatory role of TAM-derived exosomal miR-29a-3p in OC Downregulation of TAM-derived exosomal miR-29a-3p regulated the FOXO3-AKT/GSK3β axis to inhibit PD-L1 expression, ultimately inhibiting OC cell proliferation and immune escape.

In conclusion, the current study demonstrates the mechanism by which TAM-derived exosomal miR-29a-3p contributes to the development of OC, particularly regulation of the FOXO3-AKT/GSK3β signaling pathway, providing a basis supporting the potential of miR-29a-3p as a therapeutic target for OC (Figure 8). However, because of the limitations of the current experimental conditions and the limitations of the experiments themselves as well as the lack of clinical blood samples, the expression of miR-29a-3p in EVs was not detected in the blood of clinical samples, which requires further investigation.

## Materials and methods

### Ethics statement

The study protocol was performed with the approval of the Ethics Committee of The Shanghai Ninth People’s Hospital, School of Medicine, Shanghai Jiaotong University and in line with the Declaration of Helsinki. All patients and/or legal guardians provided signed informed consent documentation prior to enrollment into the study. All animal experiments were performed with the approval of the Animal Ethics Committee of The Shanghai Ninth People’s Hospital, School of Medicine, Shanghai Jiaotong University for medical research purposes. Extensive efforts were made to ensure minimal animal number use as well as suffering and pain inflicted.

### Bioinformatics analysis

The Gene Expression Omnibus (GEO) database (https://www.ncbi.nlm.nih.gov/gds) was used to identify the OC-related microarray dataset (GSE14407), which contained gene expression data and miRNA expression data. Differential analysis of the downloaded dataset was performed with the R language “limma” package, with 12 normal ovarian epithelial cell samples and 12 ovarian surface epithelial cell samples in the GSE14407. Through the miRDB (http://mirdb.org/), TargetScan (http://www.targetscan.org/vert_71/), starBase (http://starbase.sysu.edu.cn/), and mirDIP (http://ophid.utoronto.ca/mirDIP/index.jsp) databases, miRNAs that potentially possessed a targeting relationship with FOXO3 were predicted.

### Tissue sample collection

A total of 75 OC patients (age from 44 to 71 years with a mean age of 55 years) were recruited for the purposes of this study from the Shanghai Ninth People’s Hospital, School of Medicine, Shanghai Jiaotong University between January 2012 and January 2015. OC tissues (the primary lesion of OC patients) and tumor-adjacent tissue samples were collected from the patients (non-tumor tissues > 5 cm from the outer edge of the tumor in the same patient). Among the OC patients, 37 cases were epithelial OCs, 22 were serous OCs, 11 were mucinous OCs, and 5 were undifferentiated carcinomas. As per the FIGO staging system, 10 patients were at stage I, 14 patients were at stage II, 43 patients were at stage III, and 8 patients were at stage IV. The clinicopathological information of the OC patients is depicted in Table S1. None of the patients had received any chemotherapy, radiotherapy, or any other treatment prior to sample collection. All sections were diagnosed by two experienced pathologists. Patients with primary tumors <1 cm were excluded from the study. All the collected tissues were divided into two parts: one was immediately stored in liquid nitrogen, and the other was fixed in 10% formaldehyde and embedded in paraffin for sectioning. All patients were followed up for a period of 60 months, with their survival analyzed with the Kaplan-Meier method. During the follow-up period, the death of the patients was regarded as the study endpoint. If no death event occurred, the final follow-up time was recorded as the endpoint. The time interval from the date of operation to the date of death was defined as the overall survival. The number of the clinical trial was ChiCTR2000040945.

### IHC

Paraffin-embedded tissue samples in each group were sectioned (4 μm thick), deparaffinized into water, and operated on with conventional IHC staining methods with primary antibodies of anti-rabbit antibodies against FOXO3 (K001577P, 1:100, Beijing Solarbio Science & Technology Co. Ltd., Beijing, China) and PD-L1 (ab228415, 1:500, Abcam, Cambridge, UK) as well as secondary antibody against IgG (ab150083, 1:100, Abcam). Five high-power fields (100 cells per field) of each tissue section were randomly selected. The criteria for staining results were as follows: positive cells/all tumor cells > 10% was considered positive (+), and positive cells/all tumor cells ≤ 10% was considered negative (−). Sections with >50% positive cells were considered to be strongly positive. The results were independently evaluated by two experienced pathologists in a double-blind manner.

### Cell culture

Normal human ovarian epithelial cell line IOSE80, human OC cell lines (A2780, SKOV3, ES2, and COV504), and a human monocyte macrophage cell line (THP-1) were purchased from the Shanghai Meiyan Biotechnology Co., Ltd. (Shanghai, China). The used complete medium was centrifuged at 100,000 × *g* at 4°C overnight in order to remove the EVs from the medium.^29^ After recovery, all cells were cultured in RPMI 1640 medium (31870082, Gibco, Carlsbad, CA, USA) containing 10% FBS (26140079, Gibco, Carlsbad, CA, USA) and 1% double antibody (100 units/mL penicillin and 100 μg/mL streptomycin) in an incubator with a saturated humidity (BB15, Thermo Fisher Scientific, Rockford, IL, USA) at 37°C with 5% CO_2_. The medium was changed every 24 h, with the cells detached with 0.25% trypsin every 72 h for passage purposes.

TAMs were isolated from the human OC tissues by means of density gradient centrifugation for subsequent experiments.^16^^,^^30^ The THP-1-EVs were regarded as the control group, and the TAM-EVs were regarded as the experimental group.

### Lentiviral transfection

Lentiviral plasmids expressing In-miR-29a-3p (lentivirus inhibiting miR-29a-3p expression) and In-NC (NC lentivirus) as well as lentiviral packaging kits were purchased from GeneCopoeia (Rockville, MD, USA). Forty-eight hours post co-transfection of the HEK293T cells with lentiviral transfection reagents, the virus titer (5 × 10^8^ TU/mL for In-miR-29a-3p virus and 8 × 10^8^ TU/mL for In-NC virus) was determined with a p24 ELISA kit (Cell Biolabs, Inc., San Diego, CA, USA). The TAMs were subsequently infected with the prepared lentiviral particles for 24 h and cultured for another 48 h. The stably infected cell lines were selected by puromycin (P8230, Beijing Solarbio Science & Technology Co. Ltd., Beijing, China), followed by qRT-PCR to assess the infection effect.

### Cell transfection and grouping

All the following plasmids and sequences were designed and constructed into stably transfected vectors by Genewiz Company (Beijing, China). Upon reaching 80%–90% confluence, the cells were transfected in accordance with the Lipofectamine 2000 instructions (11668-019, Invitrogen, Carlsbad, CA, USA).

SKOV3/A2780 cells were treated with PBS, the supernatant of EV-free medium, TAM-EVs, TAM-In-miR-29a-3p-EVs, NC-mimic, miR-29a-3p mimic, miR-29a-3p inhibitor, NC-inhibitor, sh-NC, sh-FOXO3, oe-NC, oe-GSK3β, In-NC, or In-miR-29a-3p. In addition, SKOV3/A2780 cells without any treatment were set as the control. TAMs were infected with lentivirus expressing In-NC and In-miR-29a-3p, followed by extraction of their EV.

Co-culture was performed according to the following: 50 μg/mL TAM-In-miR-29a-3p-EVs were co-cultured with 1 × 10^5^ SKOV3 or A2780 cells with the procedures described in Flow cytometry below. In addition, the knockdown inefficiency of the two short hairpin RNAs (shRNAs), sh-FOXO3-1 and sh-FOXO3-2, were detected with western blot methods.

### Isolation and identification of EVs

EVs were removed from the medium via ultracentrifugation in order to ensure exclusion of interference. When the confluence of TAM reached 80%–90%, the cell culture supernatant was collected, after which the EVs were separated via ultracentrifuge: the collected culture supernatant was centrifuged at 500 × *g* for 15 min for cell debris removal purposes, with 2,000 × *g* for 15 min to remove the cell debris or apoptotic bodies and 10,000 × *g* for 20 min to remove large vesicles. The precipitate was then collected by centrifugation at 110,000 × *g* for 70 min after filtration with a 0.22-μm filter, resuspended with PBS followed by centrifugation at 110,000 × g for 70 min, and subsequently resuspended with 100 μL of sterile PBS for the following experiments. All ultracentrifugation steps were performed at 4°C with a Beckman (TL-100, USA) ultracentrifuge with a TLS-55 swinging bucket rotor. Low-speed centrifugation was performed with a Beckman AllegraX-15R benchtop centrifuge (USA). The EVs collected were stored at −80°C for future use.

EVs were identified and photographed by transmission electron microscopy. The particle size of EVs was analyzed with Nanoparticle Tracking Analysis (NS300, Malvern Instruments Ltd., Worcestershire, UK).^31^^,^^32^ The EV nanoparticle tracking analysis was conducted with a NanoSight NS300 instrument (Malvern Instruments Ltd., Worcestershire, UK) that was calibrated with 100-nm polystyrene beads (Polysciences, Warrington, PA, USA). The Nanoparticle Tracking Analysis was conducted with particle suspensions diluted in PBS to 1–8 × 10^8^ particles/mL. The size distribution and number of particles within the sample were determined by Stokes-Einstein equation. Western blot analysis was performed to identify the surface markers of the EVs. The protein content of EVs was determined with a bicinchoninic acid (BCA) kit (23227, Thermo Fisher Scientific, Rockford, IL, USA). The protein of the EVs was separated by sodium dodecyl sulfate polyacrylamide gel electrophoresis (SDS-PAGE) and subsequently transferred onto a new membrane with the expression of EV-specific marker protein tumor susceptibility gene 101 (TSG101) (ab30871, 1:1,000), CD63 (ab68418, 1:1,000), CD81 (ab109201, 1:2,000), and NC GRP94 (ab3674, 1:3,000) detected. Ponceau red was used as the loading control. The above antibodies were all purchased from Abcam (Cambridge, UK).

### Uptake of EVs

Purified TAM-EVs were labeled with a PKH67 green fluorescence kit (PKH67GL-1KT, Sigma-Aldrich). EVs were resuspended in 1 mL of Diluent C, and 4 μL of PKH67 ethanol dye solution was added to 1 mL of Diluent C to prepare a 4 × 10^−6^ M dye solution. A total of 1 mL of EV suspension was mixed with the dye solution for 5 min, with the staining terminated after incubation with 2 mL of 1% BSA for 1 min. Labeled EVs were ultracentrifuged at 100,000 × *g* for 70 min, washed with PBS, and ultracentrifuged again, followed by resuspension in 50 μL of PBS. PKH67-labeled EVs were incubated with OC cells for 12 h at 37°C, fixed in 4% paraformaldehyde, and washed with PBS, after which the nuclei were stained with 4′,6-diamino-2-phenylindole (DAPI). The uptake of the labeled EVs by the OC cells was determined by confocal microscopy (LSM 800, Carl Zeiss, Heidenheim, Germany).

The uptake of the TAM-derived exosomal Cy3-miR-29a-3p by OC cells was performed according to the following: TAMs were infected with Cy3-miR-29a-3p lentivirus (Shanghai GenePharma Co. Ltd., Shanghai, China) using a Lipo3000 kit (L3000001, Invitrogen, Carlsbad, CA, USA) in serum-free medium for 6 h and subsequently cultured in renewed medium containing 10% EV-free serum medium for an additional 48 h. The cell supernatants were collected, resuspended in PBS as per above-described centrifugation of EVs, and added to the OC cells. The cells were fixed with 4% paraformaldehyde, washed in PBS, and stained in an identical manner as previously mentioned, with the uptake of the TAM-derived exosomal Cy3-miR-29a-3p (red light) by OC cells observed by fluorescence microscopy (LSM710, Carl Zeiss, Heidenheim, Germany).

### CCK8 method

Cells exhibiting logarithmic growth were seeded into 96-well plates at a density of 5 × 10^4^ cells/well and incubated overnight. Cell proliferation was determined with a CCK8 kit (E606335, Shanghai Sangon Biotechnology Co. Ltd., Shanghai, China). After 0, 24, 48, and 72 h of cell culture, 10 μL of CCK8 reagent was added to each well, respectively. After incubation for an hour in a humidified incubator at 37°C, the optical density (OD) was measured at a wavelength of 450 nm with an Epoch Microplate Spectrophotometer (Omega Bio-Tek, Norcross, GA, USA). Three replicate wells were set for each group.

### Isolation of CD8^+^ T cells

The reagent for the isolation of the CD8^+^ T cells was purchased from STEMCELL Technologies (Vancouver, BC, Canada). CD8^+^ T cells were isolated after the addition of an appropriate amount of isolated antibody to the ascites of OC patients^16^ and placed at room temperature for 20 min, followed by mixing with an equal volume of PBS solution + 2% FBS. The liquid obtained was subsequently dispersed in a uniform fashion using an equal volume of lymphocyte separating medium and centrifuged at 241.5 × *g* for 20 min at room temperature. The target cells were then removed with a pipette and washed 3 times. The target cells determined to have a purity > 90% based on flow cytometry findings were used for subsequent experiments.

### Flow cytometry

#### Establishment of Transwell co-culture system

SKOV3/A2780 cells in logarithmic proliferation phase were detached with 0.25% trypsin, resuspended into single-cell suspension in McCoy’s 5A medium containing 10% FBS, and plated into 24-well plates at 2 × 10^5^ cells/well. After immunomagnetic bead sorting, CD8^+^ T cells were obtained. A Transwell co-culture system was subsequently established, after which a Transwell co-culture experiment was performed on a 24-well plate with 0.4-μm microwells. Cells were resuspended in RPMI 1640 culture medium containing 10% human AB serum. SKOV3/A2780 cells were cultured in the outer chamber of the 24-well plate, with the CD8^+^ T cells collected from healthy individuals added to the inner Transwell chamber at 6 × 10^5^ cells/well for incubation in a 5% CO_2_ incubator at 37°C. The setting of CD8^+^ T cells in the inner chamber without SKOV3 and A2780 cells in the outer chamber was used as the control group. Corresponding treatment was performed in the rescue experiment based on the aforementioned co-culture system in accordance with the experimental design. CD8^+^ T cells were collected from each group after 5 days of co-culture. Flow cytometry was used to determine the levels of immunosuppressive Treg cell-related markers (CD25, Foxp3, and CD28) in the CD8^+^ T cells of each group. Annexin V staining was used to detect the apoptotic rate of CD8^+^ T cells.

#### Detection of PD-L1 molecule on CD8^+^ T cells by flow cytometry

CD8^+^ T cells were cultured in RPMI 1640 medium containing 10% fetal calf serum (FCS) in an incubator containing 5% CO_2_ at 37°C. Cells at the logarithmic growth phase were detached with 0.25% trypsin, centrifuged, and collected, after which they were washed twice with PBS buffer containing 3% FCS and centrifuged at 1,800 rpm for 5 min at 4°C each time with the supernatant discarded. Two microliters of PE-labeled anti-human PD-L1 antibody was added to the experimental group, with the PE-labeled isotype control antibody subsequently added to the control group followed by incubation at 4°C for 15 min. After a series of PBS washes, flow cytometry was performed for detection purposes.

#### Apoptosis detection

One hundred microliters of 1× Annexin V Binding Solution was used to prepare cell suspension. Propidium iodide (PI) single-stained cells were first fixed with 75% alcohol for 30 min and then added with 5 μL of PI Solution. Single-stained cells were added with 5 μL of AnnexinV-FI, while 5 μL of AnnexinV-FI and 5 μL of PI Solution were added to the double-stained T cells, respectively. Cells in each group were cultured under dark conditions at room temperature for 15 min and added with 400 μL of Annexin V Binding Solution for testing.

### Western blot analysis

Total protein was extracted by lysing the cell lines or frozen tissue samples on ice with Radio-Immunoprecipitation assay (RIPA) lysis buffer (R0010, Solarbio) supplemented with protease inhibitor cocktail (Roche Diagnostics GmbH, Mannheim, Germany). The protein concentration of each sample was determined with a BCA kit (Pierce, Rockford, IL, USA). Total protein (30 μg) was separated by 10% SDS-PAGE and transferred to a polyvinylidene fluoride membrane (Merck Millipore, Billerica, MA, USA). The membrane was blocked in 5% skim milk for 1 h, washed three times with Tris-buffered saline Tween-20 (TBST), and then incubated with specific primary antibodies overnight at 4°C. After three TBST washes, the membrane was incubated with the corresponding secondary antibody followed by three TBST washes, with the specific bands detected with Supersignal West Pico Chemiluminescent Substrate (Thermo Fisher Scientific, Rockford, IL, USA). Glyceraldehyde-3-phosphate dehydrogenase (GAPDH) served as an internal reference. The relative content of the target proteins was expressed as the gray scale of the target protein bands relative to the gray scale of the internal reference protein bands. The main antibodies used were as follows: primary antibodies of rabbit antibodies against FOXO3 (K001577P, 1:2,500), AKT (ab179463, 1:10,000), p-AKT (Ser473) (ab81283, 1:5,000), GSK3β (ab32391, 1:5,000), p-GSK3β (Ser9) (ab107166, 1:5,000), CD68 (ab213363, 1:1,000), CD206 (ab64693, 1:1,000), and PD-L1 (ab228415, 1:1,000), secondary antibody against IgG (ab6721, 1:5,000), as well as GAPDH, which was employed as an internal reference (ab9485, 1:2,500). FOXO3 antibody was purchased from Solarbio, with the remaining antibodies purchased from Abcam.

### qRT-PCR

Total RNA was extracted from cell lines and frozen tissue samples with TRIzol reagent (15596-018, Beijing Solaibao Technology Co., Ltd., USA) as per the manufacturer’s instructions and synthesized into complementary DNA (cDNA) with the PrimeScript RT-PCR kit (TaKaRa, Mountain View, CA, USA). To detect miRNA levels, RT was performed with the PrimeScript miRNA RT-PCR kit (RR014A, TaKaRa, China) following the manufacturer’s instructions. qRT-PCR was performed on a LightCycler 480 system (Roche Diagnostics GmbH, Mannheim, Germany) with SYBR Premix Ex Taq (TaKaRa, Mountain View, CA, USA). β-Actin and U6 were employed as internal references. The primers used for amplification were purchased from Shanghai General Biotechnology Co., Ltd., with the primer sequences used depicted in Table 1. The relative transcription level of the target genes was calculated with the 2^−ΔΔCT^ method. The median expression was used to distinguish between the miR-29a-3p high-expression group (including the median) and the low-expression group.

**Table 1:** Primer sequences

Gene	Sequence
miR-29a-3p-F	5′-GGGTAGCACCATCTGAAAT-3′
miR-29a-3p-R	5′-CAGTGCGTGTCGTGGAGT-3′
FOXO3-F	5′-TCACGCACCAATTCTAACGC-3′
FOXO3-R	5′-CACGGCTTGCTTACTGAAGG-3′
PD-L1-F	5′-TCACTTGGTAATTCTGGGAGC-3′
PD-L1-R	5′-CTTTGAGTTTGTATCTTGGATGCC-3′
β-actin-F	5′-GGCCCAGAATGCAGTTCGCCTT-3′
β-actin-R	5′-AATGGCACCCTGCTCACGCA-3′
U6-F	5′-GCTTCGGCAGCACATATACTAAAAT-3′
U6-R	5′-CGCTTCACGAATTTGCGTGTCAT-3′

F, forward; R, reverse.

### Dual-luciferase reporter gene assay

The 3′ untranslated region (UTR) sequence of FOXO3 containing the predicted miR-29a-3p binding site was inserted into the pGL3-basic vector (Promega, Madison, WI, USA) at the XbaI restriction site downstream of the luciferase gene to generate the firefly/*Renilla* luciferase reporter vector pGL3-basic-FOXO3-3′-UTR-WT. The mutant was pGL3-basic-FOXO3-3′-UTR-MUT. HEK293T cells were seeded into 24-well plates and incubated for 24 h. Upon reaching 50%–60% confluence, the cells were co-transfected with NC-mimic/miR-29a-3p mimic and pGL3-basic-FOXO3-3′-UTR-WT or with NC-mimic/miR-29a-3p mimic and pGL3-basic-FOXO3-3′-UTR-MUT using Lipofectamine 2000. Furthermore, all groups were co-transfected with 10 ng of pRL-TK *Renilla* luciferase. After 24 h of transfection, cell lysates were collected to detect luciferase activity. Relative luciferase activity was determined with a dual-luciferase reporter assay system (E1910; Promega Company, Madison, WI, USA) in accordance with the manufacturer’s instructions and normalized to *Renilla* luciferase activity.

### Nude mouse xenograft experiment

Twenty-four specific pathogen-free level female BALB/c nude mice (age: 4 weeks, weight: ~20–22 g) were purchased from Beijing Animal Center, Chinese Academy of Medical Sciences, for tumor formation and immune escape experiments and housed under controlled conditions with constant humidity (45%–50%) and temperature (~25°C–27°C). Disinfected water and feed were provided *ad libitum* to the mice.

For the tumor formation assay, the concentration of SKOV3 cell suspension in logarithmic growth phase was adjusted to 2 × 10^6^ cells/mL. OC xenograft models were established by subcutaneously inoculating 0.2 mL of the cell suspension into the right groin. After the detection of palpable tumors (~1 week after injection), the mice were randomly divided into three groups (8 in each group): the PBS group (model mice receiving tail vein injection of 10 μg PBS twice a week for 3 weeks), the TAM-EVs group (model mice receiving tail vein injection of 10 μg TAM-EVs twice a week for 3 weeks), and the TAM-In-miR-29a-3p-EVs group (model mice receiving vein injection of 10 μg TAM-In-miR-29a-3p-EVs twice a week for 3 weeks). Four weeks later, the mice were euthanized via intraperitoneal injection of sodium pentobarbital (150–200 mg/kg; P3761, Sigma, St. Louis, MO, USA). Tumor growth curves were constructed with 7, 14, 21, and 28 days as the time points, and the short diameter (a) and long diameter (b) of the tumor were measured with a vernier caliper, with the tumor volume calculated according to the formula π(a2b)/6. Tumor mass was weighed with a scale. The expression of related factors in tumor tissues was detected by qRT-PCR and western blot.

Immune escape was detected based on the following: after the mice were sacrificed, the positive expression of immune-related protein (PD-L1) in the tumor tissues was detected by IHC and the expression of PD-1 molecule on CD8^+^ T cells in the spleen tissues was analyzed by flow cytometry, after which the levels of the related immune factors (TNF-α, IL-6, IL-10, and TGF-β) in the peripheral blood were detected by ELISA.

### ELISA

The preserved serum of mice in each group was centrifuged at 1,509.3 × *g* for 15 min, and the contents of immune factors TNF-α, IL-6, IL-10, and TGF-β were detected in strict accordance with the operating steps of the ELISA antibodies: TNF-α (ab208348, Abcam, Cambridge, UK), IL-6 (ab222503, Abcam, Cambridge, UK), IL-10 (ab108870, Abcam, Cambridge, UK), and TGF-β (ab119557, Abcam, Cambridge, UK).

### Statistical analysis

SPSS 21.0 (SPSS, Inc, Chicago, IL, USA) was used for statistical analyses of the data in this study. Measurement data are expressed as the mean ± standard deviation. Comparison between OC tissues and tumor-adjacent tissues was performed by a paired t test. Data comparison between two groups was performed by an unpaired t test, with data comparison between multiple groups performed with one-way analysis of variance (ANOVA) followed by Tukey’s post hoc test. The OD values and the tumor volumes at different time points were compared with two-way ANOVA followed by Bonferroni’s post hoc test. Pearson correlation was employed to analyze the relationship between the two indicators. The survival rate of the patients was calculated with the Kaplan-Meier method, and the log-rank test was applied for univariate analysis. p < 0.05 was considered to be indicative of statistical significance.

## Supplemental information

Document S1. Table S1

Document S2. Article plus supplemental information
