# Supplementary material for: TAM-derived extracellular vesicles containing microRNA-29a-3p explain the deterioration of ovarian cancer
Source: Mol Ther Nucleic Acids. 2021 May 19;25:468–82. doi: 10.1016/j.omtn.2021.05.011 (PMC8463289; doi:10.1016/j.omtn.2021.05.011)
Supplement: Document S1. Table S1 [file mmc1.pdf]

OMTN, Volume *25*

Supplemental information

TAM-derived extracellular vesicles containing microRNA-29a-3p explain the deterioration of ovarian cancer

Lili Lu, Wanwen Ling, and Zhengyi Ruan

Supplementary table 1 Clinicopathological information of OC patients

**Clinicopathological information**		n
Age		
	≥55	35
	< 55	40
Histological type		
	Epithelial OC	37
	Serous OC	22
	Mucous OC	11
	Undifferentiated carcinoma	5
FIGO staging		
	I	10
	II	14
	III	43
	IV	8
